# Genetic Diversity of the Polyomavirus JC and Implications for the Pathogenesis of Progressive Multifocal Leukoencephalopathy

**DOI:** 10.3390/v18030378

**Published:** 2026-03-18

**Authors:** Michael P. Wilczek, Sebastien Lhomme

**Affiliations:** 1 Life Science, Health, and Engineering Department, The Roux Institute, Northeastern University, Portland, ME 04101, USA; m.wilczek@northeastern.edu; 2 Observational Health Data Sciences and Informatics Center, The Roux Institute, Northeastern University, Portland, ME 04101, USA; 3 Department of Chemistry and Chemical Biology, College of Science, Northeastern University, Boston, MA 02115, USA; 4 Department of Virology, Toulouse University Hospital, 31300 Toulouse, France; 5 Toulouse Institute for Infectious and Inflammatory Diseases, INSERM UMR1291-CNRS UMR5051-Toulouse III University, 31300 Toulouse, France; 6 NEO-I3D Research Group, Toulouse University Hospital, 31300 Toulouse, France

**Keywords:** JC Polyomavirus, genotype, mutations, non-coding control region, VP1, PML

## Abstract

JC Polyomavirus (JCPyV) is a non-enveloped virus with circular double stranded DNA responsible for the rare but fatal demyelinating disease known as progressive multifocal leukoencephalopathy (PML). In its host, this virus exists in two different forms: one found in the periphery, named archetype, and another found in the central nervous system, named prototype. This form usually harbors recombinations in the non-coding control region (NCCR), a key region that contains sequences regulating viral replication and containing binding sites for cellular transcription factors. This form also contains mutations in the capsid protein, especially VP1. Due to the diversity of the JCPyV, a natural polymorphism also exists between the different genotypes. In this review, we aimed to summarize the main features of the archetype and prototype strains in order to facilitate the interpretation of sequence data that are increasingly generated by new sequencing technologies. This will also help to distinguish mutations associated with the natural polymorphism from those specific to the prototype form.

## 1. Introduction

JC Polyomavirus (JCPyV) or *Human polyomavirus 2* belongs to the *Betapolyomavirus* genus within the *Polyomaviridae* family [1]. Seven genotypes (1–4, 6–8), along with numerous subgenotypes, have been identified and linked to specific geographic regions and populations [2]. The virus was named JCPyV after the patient in whom it was first described [3]; however, the full name of the patient should no longer be used [4].

The seroprevalence of JCPyV antibodies increases with age, reaching as high as 80% among individuals aged 70 years and older [5,6,7]. Although JCPyV infection remains asymptomatic in healthy people, it can invade the central nervous system (CNS), targeting astrocytes and oligodendrocytes in immunocompromised patients. The cytolytic destruction of these cells results in the rare but fatal demyelinating disease known as progressive multifocal leukoencephalopathy (PML) [8,9]. Although PML was first described in AIDS patients and patients with hematological malignancies, other predisposing diseases such as chronic inflammatory diseases, solid organ transplantation, solid neoplasms and primary immune deficiency were identified [10]. The risk of iatrogenic PML is now well established during the use of immunomodulatory drugs such as natalizumab or rituximab [11,12]. Other manifestations due to JCPyV infection have been described, such as granule cell neuronopathy, an infection primarily of granule cells of the cerebellum [13,14,15]. Encephalopathy [16,17] and meningitis [18,19] have also been reported. More recently, the description of a unilateral retinopathy related to JCPyV infection of the retinal ganglion cell layer extended the clinical manifestations due to this virus [20]. Notably, the sequence of the virus found in this compartment was reminiscent of that with neurotropism.

In this review, we aimed to describe the variations present in the JCPyV genome, focusing not only on the regulating region but also on nonstructural and structural proteins, and to analyze their implications for the pathophysiology of the infection.

## 2. Genome Organization

**Figure 1:**
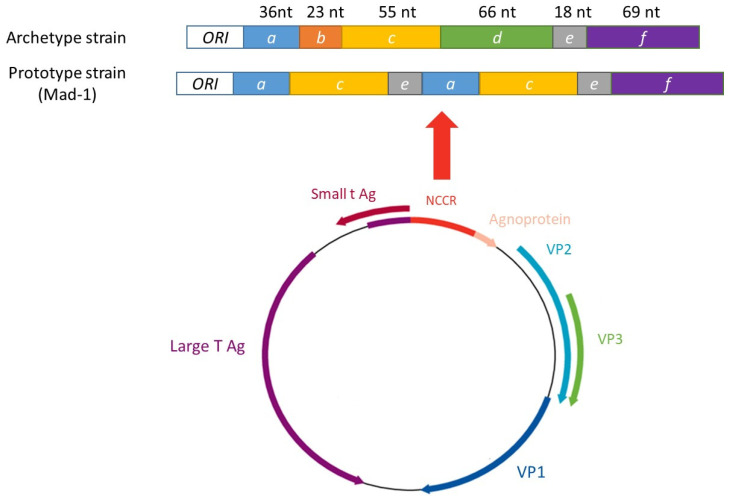
Schematic representation of the JCPyV genome.

JCPyV is a 42 nm non-enveloped virus with circular double-stranded DNA of ~5.1 kb [21]. The genome has a tripartite functional organization, including early and late regions separated by a non-coding control region (NCCR) (Figure 1). The early region encodes the non-structural proteins, including small tumor antigen (stAg) and large tumor antigen (LTAg), with TAg splice variants. These proteins increase viral transcription and a proviral environment by binding to p53, blocking the cell from activating apoptotic pathways, and can act as a helicase, unwinding the viral DNA to continue the production of virus progeny [22,23,24]. LTAg can regulate the expression of early and late genes, whereas stAg has been shown to act as an interferon antagonist. Specifically, it can interact with the E3 ubiquitin ligase TRIM25, inhibiting the ubiquitination of RIG-I and its signal transduction [25]. In vitro experiments also suggest that LTAg and stAg have oncogenic potentials [26,27].

The late region is located on the opposite DNA strand and is transcribed in the alternative direction compared to the early genes. This region encodes three capsid proteins, VP1 (354 aa), VP2 (344 aa) and VP3 (225 aa), and the non-structural agnoprotein (77 aa). VP2 and VP3 enhance the binding of LTAg to viral DNA, thereby regulating DNA replication [28]. The agnoprotein can be phosphorylated by protein kinase C [29] and dephosphorylated by protein phosphatase 2A [30]. It has been identified in the nucleus, with its phosphorylated form observed in the cytoplasm [31]. The deletion of the agnoprotein led to a significant decrease in JCPyV replication [32] and a decreased expression of both LTAg and the VP1 protein [31,32]. The NCCR, which is approximately 400 bp long, separates the early and late transcription start codons and contains sequences that regulate viral replication, as well as binding sites for cellular transcription factors [33,34,35].

The NCCR exists in two forms: a non-rearranged sequence, usually found in the urine of healthy patients. This 267 bp sequence, or the reference strain CY, is divided into six blocks in alphabetic order from *a* to *f* and corresponds to the archetype or non-diseased strain. Block *a* is 36nt, *b* is 23 nt, *c* is 55 nt, *d* is 66 nt, *e* is 18nt and *f* is 69 nt. Rearranged NCCR (rrNCCR) is usually isolated in the CSF of PML patients. The NCCR of the reference strain Mad-1, corresponding to the prototype strain, presents deletions of the block *d* and duplications of the *a*, *c* and *e* blocks [21]. Since then, many prototype strains have been described [36]. Rearrangement of the NCCR sequence seems to be a prerequisite for PML. In PML patients, the majority of JCPyV isolates had genetic mutations and rearrangements in the NCCR compared to non-disease isolates and to other regions of the viral genome, such as VP1 [37,38]. Recent research has revealed a quasispecies population within the central nervous system, where 40% of cerebrospinal fluid (CSF) sequences were identified as rrNCCR, and approximately 90% of sequences obtained from brain tissue were also rrNCCR [39]. Although the mechanisms leading to rrNCCR are unknown, it is generally acknowledged that deletions precede duplication events [40].

## 3. Mutations in the NCCR

Several studies have investigated the tissues in which these two forms are found to assess the apparent compartmentalization between them (Table 1).

In a Belgian cohort of 254 healthy subjects, 63 (24.8%) exhibited DNA shedding into their urine, with a median concentration of 6.60 log copies/mL [41]. Among the 61 sequences obtained with a Sanger method, 16 (24.6%) displayed a small deletion between 1 and 28 bp in the NCCR. Additionally, two samples contained (3.3%) insertions, specifically the duplication of 8 and 14 nt. A total of 54 urine samples from healthy people were sequenced using a 454 sequencing approach, and four samples (7.4%) contained quasispecies [41]. Variants harboring deletions never represented more than 6.5% in the quasispecies. In another study involving 400 healthy blood donors from Basel, Switzerland, 231 donors tested positive for IgG and 75 exhibited asymptomatic urinary shedding at the time of sampling. Sequences were obtained for 70 samples and a rrNCCR was found in 1/70 urine samples, with a duplication of 36 nt of the *f* block [6]. Additionally, in a cohort of 17 natalizumab-treated PML patients, Sanger sequencing demonstrated that all the NCCR sequences of the viruses isolated in the urine were archetype sequences, whereas those found in the CSF were rrNCCR [37]. Notably, the deletion of all or part of block *d* was the most frequent. A recent Indian study characterized the genetic diversity of JCPyV by sequencing the NCCR from 17 CSF of confirmed PML patients. The Sanger sequencing evidenced that deletions, duplications and insertions were frequently found in blocks *d*, *c* and *f* [50].

Delbue demonstrated this through the direct sequencing of four different NCCR sequences according to the tissue location (i.e., CSF, blood, serum and urine) from which the virus was isolated. The samples were taken from a patient living with HIV. Archetypal form (*abcdef*) was found exclusively in the urine, whereas rrNCCR was found in serum, peripheral blood cells and CSF [42]. Of note, block *d* was almost deleted in the virus isolated from the peripheral blood, and was completely deleted in the virus found in serum and CSF. This observation aligns with the hypothesis that rearrangements may occur as the virus transits from the kidney to lymphocytes [51,52].

In the study by Van Loy et al. [43], the JCPyV quasispecies was explored in 19 patients with PML using the 454-sequencing method on NCCR DNA from urine, plasma and CSF samples. A total of 10 urine, five plasma and 15 CSF samples were tested. In samples isolated from the urine, the NCCR sequence closely resembled the organization of the archetype, with the exception of one sequence that had a two bp deletion (del 221–222). In the CSF, rrNCCR sequences were found in 14 out of 15 samples. In the last sample, the viral load was significantly high (278,000,000 copies/mL) and despite the detection of archetype sequences, 12% of the quasispecies contained a deletion of block *d* and part of block *e*. Further research is needed to determine the contributions of these variants to the observed symptoms. In two patients, both plasma and CSF contained highly rearranged but identical consensus NCCR sequences.

Auvinen et al. used short-read next-generation sequencing (NGS), alongside bioinformatics tools, to characterize the NCCR in samples from 25 PML patients. They identified 11 sequences from cerebrospinal fluid (CSF) and 15 sequences from brain tissue. Archetypal NCCR was found in 2 out of 11 CSF samples, whereas archetype-like NCCR with minor mutations were found in one CSF and one brain tissue. Additionally, rrNCCR were found in 8 out of the 11 CSF and 14 out of 15 brain tissue samples [39]. Occasional JCPyV DNA detections in the brain of healthy individuals suggests that persistence of JCPyV in the brain cannot be excluded [53,54,55]. Yasuda reported a case of a 14-year-old patient with PML, where three distinct rrNCCR were found varying by brain region: the cerebellum, occipital lobe and brainstem. These findings suggest that the brain lesions may have originated independently [49].

Seppälä used a long-read sequencing technology to sequence JCPyV in the CSF of three patients with PML. One patient had an archetype-like rather than a neurotropic strain, despite mutations in the VP1 [45] (see Section 4). L’Honneur et al. used the same single-molecule sequencing technology to analyze the NCCR and VP1 sequences from 37 patients, including eight couples of CSF+ urines, 14 CSF, one cerebral biospie, 13 urine samples and for one patient, two samples of CSF [44]. Analysis of the VP1 is detailed in Section 4. Among the 24 CSF samples, deletions predominantly impacted block *d* (18/24). Other deletions affected blocks *e* and *f*, and to a lesser extent block *b*. In 21 of 24 cases, duplicated fragments (with a median size of 83 bp), mostly from sections *b* and *c*, were inserted near the *e*–*f* junction (*n* = 15) or in blocks *c* and *d*, as well as at the 5′ extremity of the late coding region (agnoprotein coding sequence). In one case, a 20-bp duplicated fragment from the *vp1* gene was inserted in NCCR. No archetypal sequence was found [44]. When analyzing the quasispecies in the CSF, only one NCCR variant was identified in 14 of 23 samples, whereas nine samples contained two, three, or more NCCR variants (in five, two, and two cases, respectively). In seven of the nine cases, the mixed population consisted of a highly predominant variant (>90%), accompanied by minor subpopulations. One patient had two CSF samples drawn at 19-day intervals and the second sample revealed an additional NCCR variant. This could be due to an increase in the viral load (from 4.2 Log copies/mL to 5.0 Log copies/mL).

This is in line with the findings of Nakamichi et al., who studied the NCCR sequences of JCPyV of CSF specimens collected from 11 immunocompromised patients. Among the four patients with an increase in the viral load during the follow-up period (median of 48.5 days between the two CSF samples), a change in the dominant NCCR pattern was observed. Conversely, the four patients with a decrease of the viral load following mefloquin administration maintained the same dominant NCCR pattern [46]. This was also observed in a patient with AIDS-related PML, following a combination of antiretroviral therapy, achieved a 99% decrease in CSF viral load [46]. Additionally, Van Loy et al. reported a case in which a single patient had three samples taken over approximately two months, during which the consensus sequence remained identical, whereas the viral load decreased [43]. Taken together, this study suggests that variants are generated de novo during viral replication or when spreading within the brain.

Iannetta reported a case of a 51-year-old PML patient living with HIV-1 in whom the archetype form of the virus was found in the CSF at t0 and t3, whereas rrNCCR was found at t1 and t2, including a duplication of the region c [47].

JCPyV was found in the CSF of a patient with multiple sclerosis treated with dimethylfumarate at a concentration of 1 988 880 copies/mL [48]. The virus found was archetype-like with a deletion in the f region.

## 4. Mutations in VP1

VP1 mediates virion binding to the cellular receptor Lactoseries tetrasaccharide c (LSTc). Consequently, mutations occurring in this protein could alter the interaction [56]. Previous studies have identified genotype-specific mutations (Table 2). In VP1, mutations at position 117 and 164 were found to be genotype specific, whereas 55, 64, 74, 75, 113, 222 and 269 were considered as sporadic [57].

**Figure 2:**
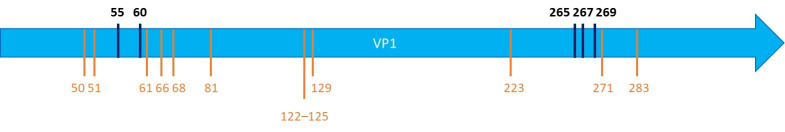
Positions of the mutations found in VP1 in JCPyV from PML patients. Positions in bold are those most frequently found to be mutated and that mediate interaction with LSTc. Other mutations were found but require further investigations.

A more detailed analysis of VP1 sequences available in GenBank, which includes 69 sequences from patients with PML, revealed that five amino acids located at positions 55, 60, 265, 267, and 269 may evolve under positive selection [58] (Figure 2). In vitro experiments using virus-like particles (VLPs) confirmed that these mutations could alter binding to sialic acid [58]. Gorelik studied the VP1 sequences from paired CSF and urine samples from eight patients [59]. The findings indicated that urine sequences were different from those found in CSF; however, plasma VP1 sequences were identical to the corresponding CSF-derived sequence [59]. This observation suggests that the virus isolated from blood and CSF acquired specific mutations in VP1 in comparison with the “parental” virus obtained from the urine. In the same study, the authors characterized the PML-associated mutations in a larger cohort of 40 patients. They found mutations or deletions in 37/40 patients, with the most common mutations at position 269 (27%) and 55 (25%). Other mutations were reported at positions 60, 61, 265, 267 and 271, as described by Sunyaev [58]. The authors also described unknown mutations at position 50, 51, 122–125 and 283. Follow-up over time indicated that mutations were maintained.

Incorporation of these mutations (55F, 60E, 265D, 267F, 269F and 269Y) in VLPs led to a loss of interaction with sialylated ganglioside, suggesting changes in receptor specificity. When tested across various cell lines, 55F, 267F, 269F and 269Y were found not to bind to kidney and lymphocytic cells but still interact with CNS cells. VLPs carrying 60E or 66H still bound to all cell types, whereas 265D or 271H VLPs bound to the kidney but not primary lymphocytes. Treatment with neuraminidase indicated that the binding to the CNS cells was independent of sialic acid [59]. The impact of these mutations on cell binding was confirmed by another group [60]. In vitro experiments using a recombinant VP1 pentamer with a single mutation in aa located in the sialic acid binding pocket of VP1 (L54F, S266F, S268F, S268Y in the original publication, likely corresponding to L55F, S267F, S269F, S269Y) indeed showed the capsid protein was no longer able to bind to LSTc [60].

In a cohort of 17 natalizumab-treated PML patients, L55 and S269 were the most observed mutations in VP1. Other mutations affecting D66, N265, S267 S61, and Q271 were found less frequently. Mutations in the VP1 were found in 13/16 (81%) of patients with PML, but not in the urine [37].

L’Honneur et al. found seven of these 12 VP1 position mutations distinctive of PML (positions 50, 51, 55, 60, 61, 122, 129, 223, 265, 267, 269, and 271; underlined positions indicate mutations that have been found). In the 23 CNS samples, Ser 269 (*n* = 10), Leu 55 (*n* = 6) and Asn 265 (*n* = 4) were the most frequently mutated. Novel mutations affecting Phe68 and Tyr81 were detected in two samples. More detailed analysis showed that four out of 23 sequences contained a 100% wildtype (wt) VP1 population, ten samples contained a virus with a single mutation affecting positions 265, 267, or 269 (7/10), or aa 55 or 61 (3/10). The other samples contained mixed subpopulations. Interestingly, ten other samples contained mixed subpopulations [44]. In three PML patients, VP1 mutations at position 267 or 8, 158, 269, 345 or 60, 69, 269 were found [45], with position 158 likely corresponding to a genotype specific signature.

Apart from PML, few studies have focused on mutations affecting VP1. C terminus mutants with a deletion of 10nt were reported during JCPyV granule neuropathy. In vitro experiments showed that such mutants are still replication competent [61].

## 5. Other Mutations

Genotype-specific variations were also reported for the early proteins tAg and Tag and the late proteins VP2, VP3 and the Agnoprotein [57,62]. Using six prototypic and ten archetype complete DNA sequences, Kato [57] showed that within LTAg, the amino acids at positions 280, 301, 354, 408, 493 and 670 vary depending on the strain’s genotype. Conversely, unique amino acid substitution could occur at position 49, 160, 233, 295, 363, 413, 479, 481, 637 and 659. In stAg, position 121 is genotype specific, whereas position 49 was variable (Table 3). In agnoprotein, positions 53 and 61 correlated to genotypes, whereas positions 32, 38 and 55 were sporadic. Some positions are both sporadic and specific. In VP2/3, eight positions were genotype specific: 175, 192, 209, 214, 253, 268, 279 and 281 (Table 4). The authors concluded that there was no specific mutation in the proteins associated with PML. One year later, Cubitt made the same analysis based on 100 sequences of JCPyV strains [62]. In L’Honneur’s work, despite the majority of reported mutations in the VP2 protein being due to genotype specificity, the authors identified novel genotype-independent mutations (A20T, A30V, A36T, and P65A) in four of 24 samples. Additionally, three missense mutations (Y407N, M402I, G255K) were found to affect the helicase domain, whereas one mutation (N299T) impacted both the Pol alpha binding domain and the DNA binding (Zn finger) domain of LTAg, which plays a crucial role in viral replication [44].

Two mutations in the LTAg coding region were found, T125A and C560S, in a cohort of 17 natalizumab-treated PML patients [37]. The use of deep sequence brought new light to JCPyV quasispecies. Takahashi evidenced that a A3495C nucleotide mutation led to a V392G aa substitution in the LTAg in all the six CSF samples from PML patients [63]. This variant represented 3% to 19% of the quasispecies. In vitro experiments indicated that introducing this mutation into the Mad1 strain decreased the expression of JCPyV proteins and DNA concentration in supernatants after the transfection of human neuroblastoma IMR-32 and human embryonic kidney HEK293 cells. This suggests that the V392G mutation in the LTAg inhibits JCPyV replication by inducing a conformational change in TAg, thereby preventing its optimal function.

JCPyV encephalopathy is due to productive infection of the cortical pyramidal neurons. In this context, Dang reported a unique JCPyV, named JCVPN1, in a non-HIV patient that presents a deletion of 143nt in the gene encoding the agnoprotein, leading to a truncated 10 amino acid peptide. The NCCR was archetype-like. The authors hypothesized that a virus expressing truncated agnoprotein could invade and accumulate in cortical pyramidal neurons [64], whereas those expressing the full agnoprotein remain mainly in glial cells. The same authors identified another variant with a different pattern of agnoprotein deletion in the CSF of a HIV+/PML patient. The impact of these strains with deletion of the agnoprotein deserves further investigation.

## 6. Consequences on the Pathophysiology of PML

Rearrangements in the NCCR were largely documented in patients suffering from PML. However, the mechanisms leading to such rearrangements and the compartment(s) where such variants emerge are largely unknown. Gosert et al. [65] showed that substitution of the archetype NCCR by a rrNCCR characterized in JCPyV isolated from the CSF of PML patients increased the replication rates in human progenitor astrocytes and human glioma cell line Hs683, but not in COS-7 cells, a fibroblast-like cell line derived from African green monkey kidney tissue constitutively expressing the SV40 LTAg. The use of a bidirectional reporter vector pHRG [66] evidenced that the rrNCCR increased early reporter gene expression. Interestingly, the deletion of block *d* led to an increased expression of the early genes. Of note, the HIV tat protein can enhance early gene expression for strains with an archetype NCCR [65]. Taken together, these results evidence that the rrNCCR could increase the early gene expression in infected cells, explaining higher replication rates. Although it is generally accepted that most rrNCCRs generate non-viable viruses, in certain instances, these rearrangements can endow the virus with new tissue tropism. In the rrNCCR, the number of transcription factor binding sites are higher due to duplication or creation compared to the archetype NCCR, notably block c duplications [36]. For instance, NFIX is known to activate JCPyV transcription [67]. Interestingly, NFIX binding sites are found in block *c* and this block is frequently duplicated in rrNCCR [36]. Conversely, the deletion of block *d* is frequent in rrNCCR. The partial or full deletion of block *d* leads to a decrease in CEBPβ binding sites, a repressor of viral transcription, ultimately leading to the enhancement of viral transcription [68]. Rearrangements in the NCCR consequently appear essential to improve the JCPyV lifecycle and to broaden the tropism of JCPyV. It is possible that the selection of adapted variants to the CNS occurs before the onset of PML.

Mutations occurring in the VP1 region during the pathogenesis of PML were also well studied [56]. Some variants seem to be associated with higher susceptibility or aggressive disease [69,70]. However, these mutations could be due to the genotype rather than a specific mutation associated with rapid or slow PML progression. This raises the question of a potential link between neuropathogenicity and genotypes [71]. The consequences of these mutations on the pathophysiology remains to be fully elucidated. The point mutations in the VP1 region could contribute to immune evasion but also affect interaction with the cellular receptors [56], including those at the surface of the neurovascular pericytes [72]. The loss of receptor interaction can be counterbalanced by the fact that the virus infects the choroid plexus, it leads to the release of extracellular vesicles containing viral particles, which may aid in dissemination within the CNS [73,74]. Consequently, alternative mechanisms of entry exist.

Mutations in other proteins are less well documented but could contribute to the improvement of the JCPyV lifecycle in infected cells.

## 7. Conclusions

In this review, we provided an overview of the mutations that have already been documented to help researchers interpret sequencing data. This is especially important for distinguishing whether mutations result from genotype polymorphisms or are linked to the emergence of a more aggressive variant during PML. NCCR and VP1 variations are well characterized. With the advent of new sequencing approaches, we can document variations in other proteins, such as TAg, tAg, Agnoprotein, VP2 and VP3. These data can lead to the identification of virological factors influencing the disease’s outcome.

## Figures and Tables

**Table 1:** Summary of JCPyV NCCR Mutations in Clinical Samples.

Arch. NCCR (%)	rr-NCCR (%)	Sequences Isolated (N)	Study	Number of Patients (N)	Specific Mutations	Key Findings
A. Urine samples from Healthy Subjects, 86% Archetype (N = 131)						
72%	28%	61	Van Loy et al., 2013 [41]	254	Deletions (1–28 bp) in 24.6%; Insertions (8–14 nt) in 3.3%	7.4% contained quasispecies (4/54)
99%	1%	70	Egli et al., 2009 [6]	400	Single rrNCCR with 36 bp duplication of f block	75/231 IgG+ donors had asymptomatic urinary shedding
B. Urine samples from PML Patients, 92% Archetype (N = 37)						
100%	0%	5	Reid et al., 2011 [37]	17	abcdef organization	Natalizumab treated cohort
100%	0%	1	Delbue et al., 2005 [42]	1	abcdef organization	HIV + PML
100%	0%	10	Van Loy et al., 2015 [43]	19	One sequence with minor 2 bp deletion (del 221–222)	NCCR closely resembled archetype
86%	14%	21	L’Honneur et al., 2022 [44]	32	Short insertions/deletions (2–38 nt); rrNCCR had deletions at 144–158 (15 bp)	PML +/− HIV- mixed population observed
C. Urine samples from SLE Patients, 50% Archetype (N = 2)						
50%	50%	2	Auvinen et al., 2024 [39]	2	Minor deletions in d block	Urine samples from two SLE patients
D. CSF samples from PML Patients, 6% Archetype (N = 136)						
0%	100%	11	Reid et al., 2011 [37]	17	Deletion of all or part of block d (most frequent)	Complete separation from urine
0%	100%	1	Delbue et al., 2005 [42]	1	Deletion: block d; duplications: blocks c and e	Progressive rearrangement
7%	93%	15	Van Loy et al., 2015 [43]	19	Deletions mostly at 61–133 bp	Highly diverse patterns
14%	86%	21	Auvinen et al., 2024 [39]	25	Partial deletion of blocks c and d	2 archetype + 1 archetype-like
20%	80%	5	Seppälä et al., 2017 [45]	3	Deletions in d block at Sp-1 binding site	Archetype-like with VP1 mutations
0%	100%	24	L’Honneur et al., 2022 [44]	32	Block d deletions (18/24); duplications of c block; insertions at the e-f junction	No archetype sequences found
0%	100%	54 ***	Nakamichi et al., 2013 [46]	11	D block deletions; duplications: blocks b, c, and e	Highly variable patterns
50%	50%	4	Iannetta et al., 2013 [47]	1	Duplication of region c in rrNCCR forms	Temporal variation (arch: t0, t3; rrNCCR: t1, t2)
100%	0	1	Motte et al., 2018 [48]	1	Deletion in f region	Patient with MS, Archetype-like; viral load 1,988,880 copies/mL
E. Blood/Peripheral Blood Samples, 0% Archetype (N = 16)						
0%	100%	9	Reid et al., 2011 [37]	17	Not specified	All contained rearranged sequences
0%	100%	2	Delbue et al., 2005 [42]	1	Partial duplication of c block; partial deletion of d block	Intermediate rearrangement pattern
0%	100%	5	Van Loy et al., 2015 [43]	19	Deletions at 115–183 bp	Less diverse than CSF
F. Brain Tissue Samples, 11% Archetype (N = 19)						
12%	88%	16	Auvinen et al., 2024 [39]	25	Partial deletion of blocks c and d	1 archetype + 1 archetype-like
0%	100%	3	Yasuda et al., 2003 [49]	1	Three distinct mutations by brain region	Independent lesion origins suggested

Abbreviations: CSF, cerebrospinal fluid; HIV, human immunodeficiency virus; MS, multiple sclerosis; NCCR, non-coding control region; PML, progressive multifocal leukoencephalopathy; rrNCCR, rearranged NCCR; t0–t3, time points; *** Nakamichi study has 54 Accession Number IDs on NCBI. Note: The archetype NCCR contains six blocks (a–f) in alphabetical order, whereas rearranged NCCR (rrNCCR) contains deletions, duplications, or rearrangements of these blocks. The block d deletion appears to be the most common rearrangement associated with neurotropism.

**Table 2:** Genotype-specific polymorphism of JCPyV VP1 protein.

	VP1 Amino Acid Variations													
**Genotype**		**11**	**37**	**73**	**74**	**112**	**116**	**127**	**133**	**157**	**163**	**320**	**331**	**344**
	**1**	**D**	**I**	**N/S**	**R**	**I**	**S**	**T/A**	**G**	**L**	**K**	**V**	**E**	**K**
	**2**	**D**	**I**	**N**	**K**	**I/L**	**T/A**	**T/A**	**G**	**V**	**V/T**	**V/I**	**E**	**R**
	**3**	**D**	**I**	**N**	**K**	**I/L**	**T**	**T**	**A**	**V**	**T**	**I**	**Q**	**R**
	**4**	**D**	**I**	**N**	**K**	**I**	**T**	**T**	**A**	**V**	**T**	**V**	**E**	**R**
	**6**	**D**	**I**	**N**	**K**	**I**	**T**	**T**	**G**	**V**	**T**	**V**	**E**	**R**
	**7**	**D**	**I/V**	**N**	**K**	**I/L**	**T**	**T**	**G**	**V**	**T**	**V**	**E/I**	**R**
	**8**	**H**	**I**	**N**	**K**	**I**	**T**	**T**	**G**	**V**	**T**	**V**	**E**	**R**

**Table 3:** Genotype-specific polymorphism of the Large T and small t antigens.

	Amino Acid Variation	Large T Antigen																					Small t Antigen		
	Position	29	155	192	202	233	280	281	301	354	364	408	470	474	479	493	533	555	653	662	667	670	29	83	121
**Genotype**	**1 A**	**V**	**R**	**G**	**H**	**F**	**E**	**T**	**Q**	**I**	**E**	**D**	**M**	**E**	**T**	**S**	**S**	**R**	**F**	**A**	**N**	**F**	**V**	**G**	**R**
	**1 B**	**V**	**R**	**G**	**H**	**F**	**E**	**T**	**Q**	**I**	**E**	**D**	**M**	**E**	**T**	**S**	**S**	**R**	**S**	**A**	**N**	**F**	**V**	**G**	**R**
	**2 A1**	**V**	**R**	**G**	**H**	**F**	**D**	**T**	**L**	**I**	* Q	**E**	**M**	**E**	**T**	**S**	* A	**R**	F *	**A**	**H**	**Y**	**V**	**G**	**R**
	**2 A2**	**V**	**R**	**G**	**H**	**F**	**D**	**T**	**L**	**I**	**E**	**E**	**M**	**E**	**T**	**S**	**S**	**R**	**F**	**A**	**H**	**Y**	**V**	**G**	**R**
	**2 B**	**V**	**R**	**G**	**H**	**F**	**D**	**T**	**Q**	**I**	**E**	**E**	**M**	**E**	**T**	**S**	**S**	**R**	**S**	**A**	**H**	**Y**	**V**	**G**	**R**
	**2 D1**	**V**	**R**	**G**	**H**	**F**	**D**	**I**	**L**	**I**	**E**	**E**	**M**	**E**	**T**	**S**	**S**	**R**	**S**	**A**	**H**	**Y**	**V**	**G**	**R**
	**2 D2**	**V**	**R**	**G**	**H**	**F**	**D**	**T**	**L**	**I**	**E**	**E**	**M**	**E**	**T**	**S**	**S**	**R**	F *	**A**	**H**	**Y**	**V**	**G**	**R**
	**2 E**	**V**	**R**	**S**	**H**	**F**	**D**	**T**	**L**	**I**	**E**	**E**	**M**	**E**	**T**	**S**	**S**	**R**	**C**	**A**	**H**	**Y**	**V**	**G**	**R**
	**3 A**	**V**	**R**	**G**	**H**	**F**	**D**	**T**	**L**	**I**	**E**	**E**	**M**	**E**	**T**	**S**	**S**	* T	**S**	**A**	**H**	**Y**	**V**	**S**	**R**
	**3 B**	**V**	**R**	**G**	**H**	**F**	**D**	**T**	**L**	**I**	**E**	**E**	**M**	**E**	**T**	**S**	**S**	**R**	**S**	**A**	**H**	**Y**	**V**	**G**	**R**
	**4**	**I**	**R**	**G**	**H**	**F**	**E**	**T**	**Q**	**I**	**E**	**D**	**M**	**E**	**T**	**S**	**S**	**R**	**F**	**A**	**N**	**Y**	**I**	**G**	**R**
	**6**	**V**	**R**	**G**	**H**	L *	**E**	**T**	**L**	**I**	**E**	**E**	**M**	**E**	* S	**N**	**S**	**R**	**S**	**A**	**H**	**Y**	**V**	**G**	**K**
	**7 A**	**V**	**R**	**G**	**H**	**F**	**D**	**T**	**L**	**I**	**E**	**E**	**M**	**E**	**T**	**S**	**S**	**R**	**S**	**A**	**H**	**Y**	**V**	**G**	**R**
	**7 B1**	**V**	**R**	**G**	**H**	**F**	**D**	**T**	**L**	* L	**E**	**E**	**M**	**E**	**T**	**S**	**S**	**R**	**S**	**A**	**H**	**Y**	**V**	**G**	**R**
	**7 B2**	**V**	**R**	**G**	**H**	**F**	**D**	**T**	**L**	**L**	**E**	**E**	**M**	**E**	**T**	**S**	**S**	**R**	**S**	**A**	**H**	**Y**	**V**	**G**	**R**
	**7 C1**	**V**	**R**	**G**	**H**	**F**	**D**	**T**	**L**	**I**	**Q**	**E**	**M**	**D**	**T**	**S**	**S**	**R**	**S**	**A**	**H**	**Y**	**V**	**G**	**R**
	**7 C2**	**V**	* K	**G**	* Q	**L**	**D**	**T**	**L**	**L**	**E**	**E**	**M**	**E**	**T**	**S**	**S**	**R**	**S**	**A**	**H**	**Y**	**V**	**G**	**R**
	**8 A**	**V**	**R**	**G**	**H**	**F**	**D**	**T**	**L**	**I**	**E**	**E**	**M**	**E**	**S**	**S**	**S**	**R**	**S**	**T**	**H**	**Y**	**V**	**G**	**R**
	**8 B**	**V**	**R**	**G**	**H**	**F**	**D**	**T**	**L**	**I**	**E**	**E**	**I**	**E**	**T**	**S**	**S**	**R**	**S**	**A**	**H**	**Y**	**V**	**G**	**R**

An aa position with a star (*) before the aa specifies that the polymorphism is found in less than 50% of the type or subtype group. A star after the aa indicates that the aa represents more than 50%. Each amino acid has its own color.

**Table 4:** Genotype-specific polymorphism of the Agnoprotein, VP2 and VP3.

	Amino Acid Variation	Agnoprotein												VP2				VP2/VP3											
	Position	45	53	55	56	57	58	59	60	61	62	63	71	5	97	102	119	161/42	175/56	179/60	192/73	209/90	214/95	253/134	268/149	279/160	281/162	326/207	342/223
**Genotype**	**1 A**	**S**	**R**	**S**	**G**	**L**	**T**	**E**	**Q**	**T**	**Y**	**S**	**T**	**L**	**L**	**F**	**A**	**I**	**A**	**Q**	**S**	**A**	**Y**	**D**	**T**	**R**	**I**	**G**	**S**
	**1 B**	**S**	**R**	**S**	**G**	**L**	**T**	**E**	**Q**	**T**	**Y**	**S**	**T**	**L**	**L**	**F**	**A**	**I**	**A**	**Q**	**N**	**A**	**Y**	**D**	**T**	**R**	**I**	**G**	**S**
	**2 A1**	**S**	**K**	**S**	**G**	**L**	**T**	**Q**	**Q**	**T**	**Y**	**S**	**T**	**L**	**L**	**F**	**A**	**I**	**S**	**Q**	**T**	**V**	**Y**	**N**	**N**	**K**	**F**	**G**	**S**
	**2 A2**	**S**	**K**	**S**	**G**	**L**	**T**	**Q**	**Q**	**T**	**Y**	**S**	**T**	**L**	**L**	**F**	**A**	**I**	**S**	**Q**	**T**	**V**	**Y**	**N**	**N**	**K**	**F**	**G**	**S**
	**2 B**	**S**	**R**	**S**	**G**	**L**	**T**	**E**	**Q**	**R**	**Y**	**S**	**T**	**L**	**L**	**F**	**A**	**I**	**S**	**Q**	**S**	**V**	**Y**	**D**	**T**	**K**	**I**	**G**	**S**
	**2 D1**	**S**	**K**	**S**	**G**	**L**	**T**	**E**	**Q**	**K**	**Y**	**S**	**T**	**V**	**L**	**F**	**A**	**I**	**S**	**Q**	**S**	**V**	**Y**	**D**	**T**	**K**	**L**	**G**	**A**
	**2 D2**	**S**	**K**	**S**	**G**	**L**	**T**	**E**	**Q**	R *	**Y**	**S**	**T**	**L**	**L**	**F**	**A**	**I**	**S**	**Q**	**S**	**V**	**Y**	**D**	**T**	**K**	**L**	**G**	**S**
	**2 E**	**S**	**K**	**S**	**G**	**L**	**T**	**E**	**Q**	**T**	**Y**	**S**	**T**	**L**	**L**	**F**	**A**	**I**	**S**	**Q**	**S**	**V**	**Y**	**D**	**T**	**K**	**I**	**G**	**S**
	**3 A**	**S**	**K**	**S**	**G**	**L**	**T**	**E**	**Q**	**T**	**Y**	**S**	**T**	**L**	**L**	**F**	**A**	**I**	**S**	R *	**S**	**V**	**Y**	**D**	**T**	**K**	**I**	**G**	**S**
	**3 B**	**S**	**K**	**S**	**G**	**L**	**T**	**E**	**Q**	**T**	**Y**	**S**	**T**	**L**	**L**	**F**	**A**	**I**	**S**	**Q**	**S**	**V**	**Y**	**D**	**T**	**K**	**I**	**G**	**S**
	**4**	**S**	**R**	**S**	**G**	**L**	**T**	**E**	**Q**	**T**	**Y**	**S**	**T**	**L**	**L**	**F**	**A**	**I**	**A**	**Q**	**N**	**A**	**Y**	**D**	**T**	**K**	**I**	**G**	**S**
	**6**	**S**	**K**	* R	**G**	**L**	**T**	**E**	**Q**	**T**	**Y**	**S**	**T**	**L**	**L**	**F**	**A**	**I**	**S**	**Q**	**S**	**V**	**F**	**D**	**T**	**K**	**I**	**G**	**S**
	**7 A**	* R	**K**	**S**	**G**	**L**	**T**	* Q	**Q**	**T**	**Y**	**S**	**T**	**L**	V *	**F**	**A**	**I**	**S**	**Q**	**S**	**V**	**Y**	**D**	**T**	**K**	**I**	**G**	**S**
	**7 B1**	**S**	**K**	**S**	**G**	**L**	**T**	* Q	**Q**	R *	**Y**	**S**	**T**	**L**	**L**	**F**	**A**	**I**	**S**	**Q**	**S**	**V**	**Y**	**D**	**T**	**K**	**I**	**G**	**S**
	**7 B2**	**R**	**K**	**S**	**G**	**L**	**T**	**E**	**Q**	**T**	**Y**	**S**	**T**	**L**	**L**	**F**	**A**	**I**	**S**	**Q**	**S**	**V**	**Y**	**D**	**T**	**K**	**I**	**G**	**S**
	**7 C1**	**S**	**K**	**S**	**G**	**L**	**T**	**E**	**Q**	**K**	**Y**	**S**	**T**	**L**	**L**	**F**	**A**	**I**	**S**	**Q**	**S**	**V**	**Y**	**D**	**T**	**K**	**I**	**G**	**S**
	**7 C2**	**S**	**K**	* R	**G**	**L**	**T**	**E**	**Q**	**T**	**Y**	**S**	**T**	**L**	**L**	**F**	**V**	**I**	**S**	**Q**	**S**	**V**	**Y**	**D**	**T**	**K**	**L**	**G**	**S**
	**8 A**	**S**	**K**	**S**	**Del**	**Del**	**Del**	**Del**	**Del**	**Del**	**Del**	**G**	**T**	**L**	**L**	**F**	**A**	**V**	**S**	**Q**	**S**	**V**	**Y**	**D**	**T**	**K**	**I**	**A**	**S**
	**8 B**	**S**	**K**	**S**	**G**	**L**	**T**	**E**	**Q**	**T**	**Y**	**S**	* K	**L**	**L**	**L**	**A**	**I**	**S**	**Q**	**S**	**V**	**Y**	**D**	**T**	**K**	**I**	**A**	**S**

An aa position with a star (*) before the aa specifies that the polymorphism is found in less than 50% of the type or subtype group. A star after the aa indicates that the aa represents more than 50%. Each amino acid has its own color.

## Data Availability

No new data were created or analyzed in this study.

## Abbreviations

The following abbreviations are used in this manuscript:

CNS	Central nervous system
CSF	Cerebrospinal fluid
JCPyV	JC Polyomavirus
LSTc	Lactoseries tetrasaccharide c
LTAg	Large T antigen
NCCR	Non-coding control region
PML	Progressive multifocal leukoencephalopathy
rrNCCR	Rearranged non-coding control region
stAg	Small t antigen
VLP	Virus-like particle
